# Determinants of Participation in the National Cancer Screening Program Among Older Korean Women: A Cross-Sectional Study Using Nationwide Population-Based Data

**DOI:** 10.3390/healthcare14081051

**Published:** 2026-04-15

**Authors:** Jin-Hee Na, Hyo-Eun Park, Seok-Hwan Kim

**Affiliations:** 1 Department of Nursing, College of Nursing, Korea University, Seoul 02841, Republic of Korea; n2011jh@naver.com; 2 Department of Nursing, College of Health Sciences, Hanseo University, Seosan-si 31962, Republic of Korea; lisa8413@gmail.com; 3 Department of Health Information Management, Dongguk University Wise Campus, Gyeongju-si 38066, Republic of Korea

**Keywords:** older adults, health behavior, health status, National Cancer Screening Program (NCSP), public health education

## Abstract

**Highlights:**

**What are the main findings?**

- Marital status, education level, private health insurance, subjective health, dyslipidemia, and physical activity were significant predictors of participation in the National Cancer Screening Program among Korean women aged 65 years or older.
- Residence area, income level, employment status, medical coverage type, hypertension, diabetes, alcohol consumption, and smoking status were not significantly associated with participation in the screening program.

**What are the implications of the main findings?**

- Targeted outreach strategies are needed to increase screening participation among vulnerable groups, particularly unmarried and less-educated older women without private insurance.
- Health promotion programs that encourage physical activity and improve awareness of cancer screening may enhance participation in national cancer screening programs among older adults.

**Abstract:**

**Background:** The incidence and mortality rates of cancer among females aged 65 years or older in the Republic of Korea are increasing; however, the national cancer screening rate (50.4%) remains low. Therefore, this study aimed to identify predictors of participation in the National Cancer Screening Program (NCSP) among women aged 65 years or older using data from the 8th Korea National Health and Nutrition Examination Survey (KNHANES VIII, 2019–2021). **Methods:** This cross-sectional study utilized data from the 8th Korea National Health and Nutrition Examination Survey (KNHANES VIII, 2019–2021). Study variables were selected based on Andersen’s healthcare utilization model. Participation in the National Cancer Screening Program (NCSP) was defined as the dependent variable, and independent variables included predisposing, enabling, and need factors. Descriptive analyses were conducted to examine participants’ characteristics. Chi-square tests were used to assess differences in NCSP participation according to participant characteristics. Multivariable logistic regression analysis was conducted to identify factors associated with participation, with all independent variables simultaneously included in the model to adjust for potential confounding. A *p*-value of <0.05 was considered statistically significant. **Results:** A total of 2105 women aged 65 years or older were included in the analysis. Of the 2105 women aged 65 years or older, 1429 (67.9%) reported participation in cancer screening within the past two years. NCSP participation was significantly associated with being married (OR = 1.540; 95% CI: 1.263–1.879), being a middle school (OR = 1.357; 95% CI: 1.022–1.801) or college graduate or higher (OR = 2.012; 95% CI: 1.199–3.378), having private insurance (OR = 1.930; 95% CI: 1.573–2.368), average subjective health (OR = 1.332; 95% CI: 1.004–1.766), dyslipidemia (OR = 1.347; 95% CI: 1.110–1.636), and physical activity participation (OR = 1.252; 95% CI: 1.029–1.524). In contrast, urban residence, income level, being employed, medical coverage type, hypertension, diabetes, monthly drinking status and current smoking status were not statistically significantly correlated with NCSP participation. **Conclusions:** These findings highlight the need for tailored outreach strategies and health education programs targeting women aged 65 years and older to enhance participation in the NCSP and ultimately improve public health outcomes.

## 1. Introduction

Cancer has become a significant global public health issue with increasing incidence and mortality rates, accounting for 16.8% of deaths from non-communicable diseases and 22.8% of all deaths worldwide [1,2]. In 2022 alone, nearly 20 million new cancer cases and 9.7 million cancer deaths were reported globally, of which nearly half of all cases (49.2%) and most deaths (56.1%) occurred in Asia [1,2]. By 2050, the number of new cancer cases is projected to increase by 77%, reaching 35 million annually [2]. In response, the World Health Organization (WHO) has recommended the development of National Cancer Control Programs (NCCPs), public health initiatives aimed at reducing cancer incidence and mortality while enhancing quality of life [3,4]. These programs are crucial in efficiently utilizing resources to mitigate the detrimental impact of cancer on individuals and communities [3,4]. They represent the best public health strategy for decreasing cancer rates and improving survival outcomes, having markedly reduced worldwide cancer mortality [3,4]. Meanwhile, the goal of NCCPs has been reported to be to further reduce cancer mortality by up to one-third through early detection and treatment of cancer [5,6].

The Republic of Korea has highlighted the importance of early detection through its National Cancer Screening Program (NCSP), launched in 1999 [7,8,9]. Currently, the NCSP for women in South Korea covers breast cancer, cervical cancer, colon cancer, gastric cancer, liver cancer, and lung cancer [7,8,9]. Cancer screening recommendations for Korean women are as follows: breast cancer for those 40 and older, cervical cancer for those 20 and older, colon cancer for those 50 years of age and older, gastric cancer for those 40 and older, liver cancer for those 40 years of age or older (or those with cirrhosis, positive hepatitis B virus antigen or hepatitis C virus antibody), and lung cancer for those aged 54 to 74 [7,8,9]. The screening frequency for each cancer in women is 2 years for breast cancer, cervical cancer, gastric cancer, and lung cancer, 1 year for colon cancer, and 6 months for liver cancer [7,8,9]. Over the past five years, the Republic of Korea’s national cancer screening participation rates have hovered around 55% (2018: 55.0%; 2019: 55.6%; 2020: 49.2%; 2021: 55.1%) [7,8,9]. The most recently reported NCSP participation rate in 2022 was 58.2%, at 40.3% among men and 59.7% among women [7,8,9]. However, the participation rate among adults aged 65 and older was markedly lower, at 50.4% for women and 55.7% for men [7,8,9]. This falls short of the WHO’s and Forrest Report’s recommended participation rate of at least 70% to effectively reduce mortality rates through NCCPs [10,11,12]. In particular, the low participation rate of the Republic of Korea’s female elderly population in NCSP was due to retirement from work, having no spouse, low education, and lack of knowledge about cancer screening, and the burden of paying additional costs [13,14,15,16,17]. In the Republic of Korea, cancer remains the leading cause of death, with 83,378 individuals (22.4% of all deaths) dying from the disease in 2022 [7]. In fact, the cancer mortality rate has steadily increased from 146.5 per 100,000 in 2012 to 162.7 per 100,000 in 2022 [7]. The proportion of women aged 65 and older in the Republic of Korea’s population has steadily increased, from 9.1% in 2000 to 15.1% in 2015 and 17.2% in 2019 [18]. In addition, the cancer incidence rate per 100,000 women aged 65 years or older increased from 89.5 (2016) to 117.6 (2021) for breast cancer, from 130.4 (2017) to 137.2 (2021) for lung cancer, from 142.3 (2020) to 153.9 (2021) for colon cancer, and from 108.3 (2020) to 114.1 (2021) for stomach cancer [18]. Cumulatively, these results indicate that as the Republic of Korea faces rapid population aging, with a growing number of women aged 65 and older, corresponding increases are occurring in cancer incidence and mortality rates [19,20]. Notably, chronic diseases are becoming more prevalent in this population, while rates of physical activity and self-rated health remain low [19,20].

In the past decade, several studies have identified various factors affecting participation in cancer screening, including age [13,14,15,21,22,23,24,25,26,27,28,29], sex [13,26], marital status [13,15,26,30,31,32], residence [22,30,31], education level [13,15,22,25,26,31,33], income [13,22,31,33], economic activity [13,15,31], health insurance type [21,31,33], private insurance [13,15], dyslipidemia [22], diabetes mellitus (DM) [23], perceived health status [15], physical activity [13,15,22,23,25], alcohol consumption [21,22,23], and smoking status [13,15,21,23]. However, these studies have produced mixed results. Women aged 50 to 59 years [29], who were married [30], who had a higher level of education, wealth [33], and who had health insurance were more likely to receive breast cancer screening [30,33]. And women with private insurance [21], below the age of 40 years, with spouse encouragement [31], who were married, who had awareness of screening methods, who were of increasing age, and who received physician recommendation [32] were more likely to receive cervical cancer screening. Also, married women [26,34,35] and highly educated women [26] were more likely to participate in colorectal cancer screening programs.

Prior research has focused primarily on specific types of cancer screenings, with minimal attention paid to participation in NCCPs (National Cancer Control Programs) as a whole. There is also a relative lack of research targeting the predictors of participation in NCCPs among women aged 65 and older, a population identified as facing barriers to NCCP access. Therefore, this study aimed to analyze the predictors of participation in the NCSP among older women in the Republic of Korea to provide valuable insights that can inform strategies to increase screening rates and early cancer detection. Despite previous studies examining factors associated with cancer screening participation, limited research has focused specifically on older women using a comprehensive theoretical framework and recent nationally representative data. Therefore, this study aimed to identify determinants of participation in the NCSP among Korean women aged 65 years or older using KNHANES VIII data based on Andersen’s healthcare utilization model.

This study aimed to identify factors associated with participation in the National Cancer Screening Program (NCSP) among Korean women aged 65 years or older using data from the Korea National Health and Nutrition Examination Survey (2019–2021). Specifically, this study aims to:

(1) Describe the general characteristics of the participants;
(2) Assess NCSP participation patterns in relation to participants’ sociodemographic characteristics, health status, and health-related behaviors;
(3) Determine the key predictors influencing NCSP participation in the study population.

## 2. Materials and Methods

### 2.1. Data Collection

Data were extracted from the KNHANES VIII (2019–2021), a nationally representative cross-sectional survey conducted by the Korea Disease Control and Prevention Agency. In the KNHANES, data on marital status and residence were originally collected through face-to-face interviews conducted by trained survey personnel [36,37]. This survey applied a complex, stratified, multistage probability sampling design based on sex, age, and region to accurately represent the non-institutionalized civilian Korean population [36,37]. To ensure consistent and reliable performance and reduce bias in the interviews and surveys, KNHANES VIII deployed a technical investigation team comprising a nutritionist, a nurse, and a health science major, whose investigative performance was regularly verified and maintained through education and field quality control [36,37]. From the total target population of KNHANES VIII (2019–2021) (N = 29,649), individuals aged 65 years and older were first extracted. Among them, males (n = 1966) and samples with missing data (n = 1214) were excluded, resulting in a final analytic sample of 2105 participants. In KNHANES VIII (2019–2021), individual non-response was corrected using non-response adjustment weights, while item non-response was not replaced because the non-response rate was low based on the item-level distribution review [36,37].

### 2.2. Selection of Variables

Variables were selected based on Andersen’s healthcare utilization model, which categorizes determinants of health service use into predisposing, enabling, and need factors [38,39,40]. This model has been widely applied to identify factors associated with healthcare utilization [14,16,33,41,42,43,44]. Participation in the National Cancer Screening Program (NCSP) was defined as the dependent variable. Participation was operationalized as engagement in any cancer screening within the past two years, based on self-reported survey data. Independent variables were categorized according to Andersen’s model [38,39,40]. Predisposing factors included age, marital status, education level, and residence. Enabling factors included income level, economic activity, type of medical coverage, and private health insurance. Need factors included subjective health status, hypertension, diabetes, dyslipidemia, physical activity, alcohol consumption, and smoking status [38,39,40].

### 2.3. Definition of Variables

#### 2.3.1. National Cancer Screening Program (NCSP)

Trained interviewers collected responses regarding the NCSP from all participants. The questionnaire (Supplementary Materials) asked, “Have you had a cancer screening in the past two years?” and was dichotomized into yes or no [36,37].

#### 2.3.2. Sociodemographic and Health Behavior Variables

Independent variables were classified into predisposing factors, enabling factors, and need factors as in the Anderson model. A skilled interviewer surveyed all items; health behavior areas such as smoking and drinking were surveyed through self-reporting. Data on marital status and residence were obtained through face-to-face interviews. Education level was classified into four groups. The household income was calculated by standardizing monthly income according to the number of family members, and income levels were divided into four groups. Economic activity was assessed based on the question, “During the past week, have you worked for more than one hour for income or worked as an unpaid family worker for more than 18 h? Yes/No.” The medical coverage type was queried by asking, “What health insurance are you enrolled in?” To assess private insurance, the participants were asked, “Have you signed up for private health insurance that subsidizes medical expenses, such as cancer insurance, cardiovascular disease insurance, and accident insurance sold by insurance companies?” Subjective health was assessed by asking, “How do you usually feel about your health?” Responses were divided into three categories. Physical activity was defined as “a person who engages in moderate-intensity physical activity for more than 2 h and 30 min per week, or vigorous-intensity physical activity for more than 1 h and 15 min per week, or a combination of moderate-intensity and vigorous-intensity physical activity (1 min of vigorous-intensity equaled 2 min of moderate-intensity) for an equivalent amount of time per week.” Physical activity was assessed with the following questions: “Do you usually engage in high-intensity sports, exercise, and leisure activities that make you breathe heavily or your heart beat very fast for at least 10 min? (Yes/No) Do you usually engage in moderate-intensity sports, exercise, and leisure activities that make you slightly breathless, or your heart beat slightly fast for at least 10 min? (Yes/No).” Monthly drinking status was measured by asking, “Have you ever had more than one glass of alcohol in your life? How often have you consumed alcohol in the past year?”. The current smoking status was defined as “smoked more than five packs (100 cigarettes) of regular cigarettes (cigarettes) in their lifetime and currently smoke regular cigarettes (cigarettes)” [36,37].

#### 2.3.3. Biochemical Measurements

The 8th Korean National Health and Nutrition Examination Survey (KNHANES VIII) collected data through a household membership survey, health questionnaire, physical examination, and nutrition survey. The health and physical examination surveys were conducted in a mobile screening vehicle, while the nutrition survey was conducted by visiting target households in person [36,37]. In addition, hypertension (HTN) was diagnosed by measuring blood pressure through a physical examination, and diabetes mellitus (DM) and dyslipidemia were diagnosed by a blood test. Trained staff members in the Division of Chronic Disease Surveillance under the Korea Centers for Disease Control and Prevention and the Korean Ministry of Health and Welfare took the participants’ physical measurements. HTN was defined as “systolic blood pressure ≥ 140 mmHg or diastolic blood pressure ≥ 90 mmHg or taking antihypertensive medication.” DM was defined as “a non-pregnant person who has fasted for more than eight hours, has a fasting blood sugar level of 126 mg/dL or higher, has been diagnosed by a doctor, is taking hypoglycemic agents or insulin injections, or has a glycated hemoglobin level of 6.5% or higher.” Dyslipidemia included hypercholesterolemia or hypertriglyceridemia. Hypercholesterolemia was defined as “a person whose total cholesterol is 240 mg/dL or higher or who is taking cholesterol-lowering drugs.” Hypertriglyceridemia was defined as “a person whose triglyceride is 200 mg/dL or higher (applying a fasting time of 12 h)” [36,37].

### 2.4. Statistical Analysis

All statistical analyses were performed using SPSS version 27.0 (IBM Corp., Armonk, NY, USA), and a *p*-value < 0.05 was considered statistically significant. Descriptive statistics were used to summarize participants’ characteristics, including sociodemographic factors, health status, and health behaviors. Chi-square tests were conducted to examine differences in national cancer screening participation according to participants’ characteristics. Binary logistic regression analysis was performed to identify factors associated with participation in the National Cancer Screening Program (NCSP). Variables that were statistically significant in the chi-square analyses were included in the logistic regression model.

Before conducting the logistic regression analysis, key assumptions were evaluated. Multicollinearity among independent variables was assessed using variance inflation factors (VIFs), with values ranging from 1.015 to 1.277, indicating no evidence of multicollinearity. As most independent variables were categorical, the assumption of linearity of the logit for continuous variables was not applicable. The independence of observations was ensured based on the cross-sectional design of the KNHANES dataset, in which each participant represents an independent observation.

## 3. Results

### 3.1. Participants’ General Characteristics

The frequencies and general characteristics of the study participants are presented in Table 1. Among the total 2105 participants, 1429 (67.9%) underwent cancer screening. Regarding marital status, 1133 participants (53.8%) were married, and 1500 (71.3%) resided in urban areas. In terms of education level, the smallest proportion was those with a college degree or higher (114 participants, 5.4%). For income level, the high-income group accounted for 175 participants (8.3%), and 675 participants (32.1%) were employed.

Regarding types of health insurance, 175 participants (8.3%) were covered by Medical Aid, while 1930 (91.7%) were covered by National Health Insurance. A total of 1181 participants (56.1%) had private insurance. For subjective health status, those who perceived their health as “good” were the fewest, accounting for 410 participants (19.5%).

Among chronic disease characteristics, 1372 participants (65.2%) had hypertension, and 603 (28.6%) had diabetes. A total of 1105 participants (52.5%) had dyslipidemia. Regarding health behaviors, 990 participants (47.0%) engaged in physical activity, monthly alcohol consumption was reported by 335 participants (15.9%), and 47 participants (2.2%) were current smokers.

### 3.2. Factors Associated with NCSP Participation

The results of participation in National Cancer Screening Program examination according to the characteristics of the study participants are presented in Table 2. Among sociodemographic characteristics, cancer screening rates were higher among married individuals (841 participants, 74.2%; *p* < 0.001) and those residing in urban areas (1038 participants, 69.2%; *p* < 0.05). Regarding education level, the highest screening rate was observed among those with a college degree or higher (93 participants, 81.6%; *p* < 0.05), and among income groups, the upper-middle group showed the highest rate (243 participants, 75.7%; *p* < 0.001).

With respect to health insurance type, those covered by National Health Insurance had a higher screening rate (1323 participants, 68.5%) than those receiving Medical Aid (106 participants, 60.6%; *p* < 0.05). Participants with private insurance also showed a higher screening rate (896 participants, 75.9%; *p* < 0.001).

Among health status variables, individuals without hypertension had a higher screening rate (529 participants, 72.2%; *p* < 0.05), and those without diabetes showed a higher rate as well (1039 participants, 69.2%; *p* < 0.05). Participants with dyslipidemia exhibited a higher screening rate (794 participants, 71.9%; *p* < 0.001).

Regarding health behaviors, those engaging in physical activity had a higher screening rate (716 participants, 72.3%; *p* < 0.001), and non-smokers showed a higher participation rate (2590 participants, 69.9%; *p* < 0.01). However, economic activity (sociodemographic characteristics), subjective health perception (health status), and alcohol consumption and smoking behavior (health behaviors) were not statistically significant.

### 3.3. Predictors of NCSP Participation (Multivariate Analysis)

Table 3 presents the results of the regression analysis performed to identify the predictors of NCSP participation among the study population. NCSP participation was significantly associated with being married (OR = 1.540; 95% CI: 1.263–1.879), being a middle school (OR = 1.357; 95% CI: 1.022–1.801) or college graduate or higher (OR = 2.012; 95% CI: 1.199–3.378), having private insurance (OR = 1.930; 95% CI: 1.573–2.368), average subjective health (OR = 1.332; 95% CI: 1.004–1.766), dyslipidemia (OR = 1.347; 95% CI: 1.110–1.636), and physical activity participation (OR = 1.252; 95% CI: 1.029–1.524). In contrast, urban residence, income level, being employed, medical coverage type, hypertension, diabetes, monthly drinking status and current smoking status were not statistically significantly correlated with NCSP participation.

**Figure 1:**
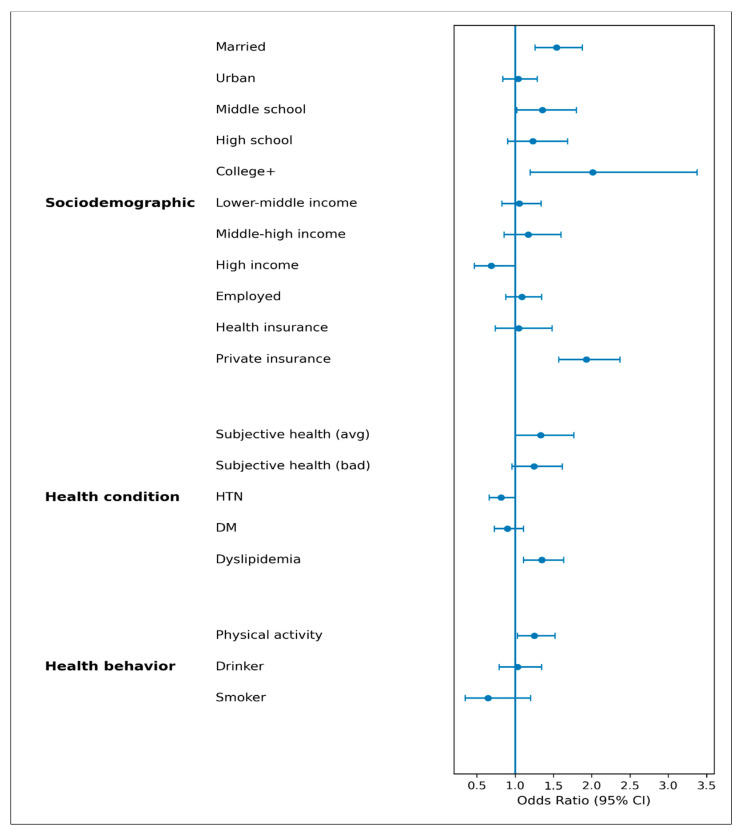
Forest plot of predictors of NCSP participation.

To examine the multicollinearity among the independent variables, variance inflation factors (VIFs) were calculated. The VIF values ranged from 1.015 to 1.277, which are well below the threshold of 10. These results indicate that there were no multicollinearity issues among the variables included in the model. The results of the multivariable logistic regression analysis are visually presented in Figure 1.

## 4. Discussion

### 4.1. Interpretation of Key Findings

This study utilized data from the 8th Korea National Health and Nutrition Examination Survey (KNHANES VIII, 2019–2021) to examine factors associated with participation in the National Cancer Screening Program (NCSP) among women aged 65 years and older.

Among sociodemographic factors, marital status was significantly associated with NCSP participation. Married participants were more likely to participate in cancer screening compared to single participants. Similar findings have been reported in previous studies conducted in the United States, where married older women showed higher adherence to colorectal cancer (CRC) screening [34,35] and breast cancer screening [35] guidelines. In Canada, marriage was also associated with increased participation in cervical cancer screening [45], possibly reflecting the role of spousal support and shared health-related behaviors [46].

Education level was also significantly associated with cancer screening participation. Participants with higher levels of education were more likely to participate in screening than those with lower educational attainment. This is consistent with studies from Taiwan, where older women with lower education levels were less likely to undergo mammography [47]. Similarly, in the United States, higher education levels have been associated with greater adherence to CRC screening guidelines [34] and higher participation in breast cancer screening [48]. Among Chinese-American older women, higher education has also been associated with increased participation in breast and cervical cancer screening [49]. Higher educational attainment may be associated with improved access to health-related information and greater awareness of preventive healthcare services.

Private health insurance was another factor significantly associated with NCSP participation. Participants with private insurance were more likely to undergo cancer screening than those without it. Similar patterns have been observed in the United States, where private insurance coverage has been associated with increased participation in CRC screening [50]. In addition, Chinese-American women aged 65 years and older with private insurance showed higher participation in cancer screening [51]. Private insurance coverage may be associated with reduced financial and psychological barriers to screening and improved access to healthcare services.

Regarding subjective health, participants who rated their health as average were more likely to participate in the NCSP than those who rated their health as good. A meta-analysis of breast cancer screening rates among older women reported that individuals with poorer perceived health were less likely to undergo mammography [52]. In France, better perceived health has been associated with higher participation in cervical, breast, and CRC screening [21]. Similarly, a study using Italian PASSI data reported higher participation in cancer screening among individuals with better perceived health status [23]. The discrepancies between previous studies and our findings may be related to differences in study populations, cultural contexts, and analytical approaches.

Among health conditions, dyslipidemia was significantly associated with higher participation in cancer screening. This finding is consistent with a study conducted in Japan, where older women with dyslipidemia showed higher participation in cancer screening [53]. A systematic review also reported that individuals with chronic conditions are more likely to participate in CRC screening [54]. In addition, Italian PASSI data indicated higher participation rates among individuals with dyslipidemia [23]. This association may reflect increased contact with healthcare providers and greater awareness of health risks among individuals with chronic conditions.

In terms of health behaviors, physical activity was positively associated with participation in cancer screening. This finding is consistent with a study conducted in Taiwan, which reported that older women who did not engage in regular physical activity were less likely to participate in national cancer screening programs [55]. In the United States, regular physical activity has also been associated with higher participation in breast and cervical cancer screening [56]. Physical activity may be associated with greater health awareness and proactive health management behaviors.

Based on these findings, several implications can be considered. Efforts to improve cancer screening participation among older women may benefit from targeted interventions focusing on individuals who are socially isolated, have lower educational attainment, or lack private health insurance. In addition, tailored health education and outreach programs may help address barriers related to perceived health status and health behaviors.

It is also important to consider that older adults in Korea may access cancer screening services through both the NCSP and private healthcare providers. Due to the limitations of the secondary data used in this study, participation in private or voluntary cancer screening programs could not be distinguished. Therefore, non-participation in the NCSP should be interpreted with caution, as it may not necessarily indicate a complete lack of cancer screening.

The NCSP has implemented various outreach strategies, including mailed invitation letters, text message reminders, and community-based promotion through public health centers, as well as initiatives to improve accessibility such as mobile screening services. Despite these efforts, participation among older women remains suboptimal. This suggests that existing strategies may not fully address barriers specific to this population, such as living alone, limited health literacy, transportation difficulties, and perceived health status. More tailored and age-specific approaches may be required to improve screening uptake in this group.

Finally, given the cross-sectional design of this study, the observed relationships should be interpreted as associations rather than causal effects.

### 4.2. Limitations

This study has several limitations. First, due to the nature of secondary data, we could not include cases in which individuals participated in other voluntary cancer screening programs. Second, geographic barriers to cancer screening facilities could not be assessed. Third, the dependent variable was based on participation in any cancer screening within the past two years and did not distinguish between specific cancer types. Therefore, cancer-type-specific analyses (e.g., breast or cervical cancer) could not be conducted. Finally, because this study employed a cross-sectional design, the observed associations cannot be interpreted as causal relationships; thus, the findings should be interpreted as exploratory rather than confirmatory. Additionally, as the data were based on self-reported responses, recall bias may have affected the accuracy of cancer screening participation.

### 4.3. Implications

This study contributes to the literature by focusing specifically on older Korean women, a population group that has been relatively underexplored despite their increasing cancer burden. In addition, unlike previous studies that examined screening for specific cancer types, this study evaluated participation in cancer screening at the program level using nationally representative data. Furthermore, this study applied Andersen’s behavioral model to comprehensively examine multiple domains of determinants, providing a more integrated understanding of factors associated with screening participation.

Future research should investigate the accessibility of the NCSP to older adults by incorporating transportation-related factors and further examine the unique characteristics of older adults living alone.

## 5. Conclusions

This study revealed that participation in the NCSP among women aged 65 years and older in the Republic of Korea was significantly affected by marital status, education level, private insurance, subjective health, dyslipidemia, and physical activity, but not by factors such as residence, income, employment, type of health insurance, HTN, DM, monthly alcohol consumption, or current smoking status. Those with very good or very poor perceived health may forgo screening due to preconceived notions about their cancer screening results. Meanwhile, those with dyslipidemia and concerns about their health who choose to engage in regular physical activity are more likely to participate in cancer screenings. These decisions are further influenced by access to private health insurance and corresponding access to healthcare services. These findings highlight the need for targeted cancer screening programs tailored to older women, in addition to measures to promote public awareness, public campaigns, clinical interventions, and management. Our findings support the WHO recommendations that increasing cancer screening among older women can reduce cancer incidence and mortality as well as improve quality of life [3,4].

Therefore, prioritizing the single group of less-educated women aged 65 years and older, as well as other disadvantaged groups, and reinforcing screening services is crucial for public health. Enhancing targeted outreach and education could facilitate participation in the NCSP and consequently improve the public’s health.

## Figures and Tables

**Table 1:** General characteristics of participants.

Variable			*n*	%
Sociodemographic characteristic	Cancer screening	Not examined	676	32.1
		Examined	1429	67.9
	Marital status	Single	972	46.2
		Married	1133	53.8
	Residence	Rural	605	28.7
		Urban	1500	71.3
	Education level	Elementary school or less	1361	64.7
		Middle school graduate	345	16.4
		High school graduate	285	13.5
		College graduate or higher	114	5.4
	Income level	Lower	1047	49.7
		Lower-middle	562	26.7
		Upper-middle	321	15.2
		High	175	8.3
	Economic activity	Unemployed	1430	67.9
		Employed	675	32.1
	Medical coverage type	Medical care	175	8.3
		Health insurance	1930	91.7
	Private insurance	Not joined	924	43.9
		Joined	1181	56.1
Health condition	Subjective health	Bad	687	32.6
		Average	1008	47.9
		Good	410	19.5
	HTN	No	733	34.8
		Yes	1372	65.2
	DM	No	1502	71.4
		Yes	603	28.6
	Dyslipidemia	No	1000	47.5
		Yes	1105	52.5
Health behavior	Physical activity	No	1115	53.0
		Yes	990	47.0
	Monthly drinking status	Non-drinking	1770	84.1
		Drinking	335	15.9
	Current smoking status	Non-smoking	2058	97.8
		Smoking	47	2.2
Total			2105	100.0

**Table 2:** Analysis of NCSP participation by participants’ characteristics.

Variable			Cancer Screening		Total	*χ* ^2^	*p*
			Not Examined	Examine			
Sociodemographic characteristic	Marital status	Single	384 (39.5%)	588 (60.5%)	972 (100.0%)	45.264 ^a^	0.000
		Married	292 (25.8%)	841 (74.2%)	1133 (100.0%)		
	Area of residence	Eup Myeon (Rural)	214 (35.4%)	391 (64.6%)	605 (100.0%)	4.133 ^a^	0.042
		Dong (Urban)	462 (30.8%)	1038 (69.2%)	1500 (100.0%)		
	Education level	Elementary school or lower	494 (36.3%)	867 (63.7%)	1361 (100.0%)	33.158 ^a^	0.042
		Middle school	88 (25.5%)	257 (74.5%)	345 (100.0%)		
		High school	73 (25.6%)	212 (74.4%)	285 (100.0%)		
		College or higher	21 (18.4%)	93 (81.6%)	114 (100.0%)		
	Income level	Low	383 (36.6%)	664 (63.4%)	1047 (100.0%)	22.341 ^a^	0.000
		Lower-middle	159 (28.3%)	403 (71.7%)	562 (100.0%)		
		Upper-middle	78 (24.3%)	243 (75.7%)	321 (100.0%)		
		High	56 (32.0%)	119 (68.0%)	175 (100.0%)		
	Employment status	Unemployed	473 (33.1%)	957 (66.9%)	1430 (100.0%)	1.897 ^a^	0.168
		Employed	203 (30.1%)	472 (69.9%)	675 (100.0%)		
	Health insurance type	Medical aid	69 (39.4%)	106 (60.6%)	175 (100.0%)	4.684 ^a^	0.030
		Health insurance	607 (31.5%)	1323 (68.5%)	1930 (100.0%)		
	Private health insurance	No	391 (42.3%)	533 (57.7%)	924 (100.0%)	78.627 ^a^	0.000
		Yes	285 (24.1%)	896 (75.9%)	1181 (100.0%)		
Health condition	Subjective health	Poor	225 (32.8%)	462 (67.2%)	687 (100.0%)	0.394 ^a^	0.821
		Average	317 (31.4%)	691 (68.6%)	1008 (100.0%)		
		Good	134 (32.7%)	276 (67.3%)	410 (100.0%)		
	Hypertension	No	204 (27.8%)	529 (72.2%)	733 (100.0%)	9.464 ^a^	0.002
		Yes	472 (34.4%)	900 (65.6%)	1372 (100.0%)		
	Diabetes mellitus	No	463 (30.8%)	1039 (69.2%)	1502 (100.0%)	3.993 ^a^	0.046
		Yes	213 (35.3%)	390 (64.7%)	603 (100.0%)		
	Dyslipidemia	No	365 (36.5%)	635 (63.5%)	1000 (100.0%)	16.809 ^a^	0.000
		Yes	311 (28.1%)	794 (71.9%)	1105 (100.0%)		
Health behavior	Physical activity	No	402 (36.1%)	713 (63.9%)	1115 (100.0%)	16.880 ^a^	0.000
		Yes	274 (27.7%)	716 (72.3%)	990 (100.0%)		
	Monthly alcohol consumption	No	577 (32.6%)	1193 (67.4%)	1770 (100.0%)	1.199 ^a^	0.273
		Yes	99 (29.6%)	236 (70.4%)	335 (100.0%)		
	Current smoking status	Non-smoker	657 (31.9%)	1401 (68.1%)	2058 (100.0%)	1.523 ^a^	0.217
		Smoker	19 (40.4%)	28 (59.6%)	47 (100.0%)		
Total			676 (32.1%)	1429 (67.9%)	2105 (100.0%)		

^a^: Adjusted for age, education, and income.

**Table 3:** Relative likelihood of NCSP participation by participants’ characteristics.

Variable			B	*p*	OR	95% CI		VIF
						Min.	Max.	
Social demographic characteristics	Marital status	Single(=ref.)	1.000					1.098
		Married	0.432	0.000	1.540	1.263	1.879	
	Residence	Rural(=ref.)	1.000					1.082
		Urban	0.040	0.718	1.040	0.840	1.289	
	Education level	Elementary school or less(=ref.)	1.000					1.229
		Middle school graduate	0.305	0.035	1.357	1.022	1.801	
		High school graduate	0.210	0.189	1.233	0.902	1.686	
		College graduate or higher	0.699	0.008	2.012	1.199	3.378	
	Income level	Lower(=ref.)	1.000					1.277
		Lower-middle	0.051	0.679	1.053	0.826	1.341	
		Upper-middle	0.157	0.323	1.170	0.857	1.599	
		High	−0.377	0.055	0.686	0.467	1.008	
	Economic activity	Unemployed(=ref.)	1.000					1.067
		Employed	0.082	0.446	1.086	0.878	1.343	
	Medical coverage type	Medical care(=ref.)	1.000					1.100
		Health insurance	0.046	0.795	1.047	0.739	1.485	
	Private insurance	Not join(=ref.)	1.000					1.174
		Join	0.657	0.000	1.930	1.573	2.368	
Health condition	Subjective health	Good(=ref.)	1.000	0.126				1.075
		Average	0.287	0.046	1.332	1.004	1.766	
		Bad	0.221	0.096	1.247	0.961	1.617	
	HTN	No(=ref.)	1.000					1.061
		Yes	−0.204	0.055	0.816	0.662	1.004	
	DM	No(=ref.)	1.000					1.044
		Yes	−0.106	0.328	0.899	0.727	1.112	
	Dyslipidemia	No(=ref.)	1.000					1.052
		Yes	0.298	0.003	1.347	1.110	1.636	
Health behavior	Physical activity	No(=ref.)	1.000					1.059
		Yes	0.225	0.025	1.252	1.029	1.524	
	Monthly drinking rate	Non-drinker(=ref.)	1.000					1.020
		Current drinker	0.030	0.826	1.030	0.789	1.345	
	Current smoking rate	Non-smoker(=ref.)	1.000					1.015
		Current smoker	−0.435	0.168	0.647	0.349	1.200	

## Data Availability

The datasets generated during and/or analyzed during the current study are available in the Korea Centers for Disease Control and Prevention repository [http://knhanes.cdc.go.kr]. The datasets generated during and/or analyzed during the current study are also available from the corresponding author upon reasonable request.

## Supplementary Materials

The following supporting information can be downloaded at: https://www.mdpi.com/article/10.3390/healthcare14081051/s1, Questionnaire.

## Abbreviations

The following abbreviations are used in this manuscript:

CRC	Colorectal Cancer
DM	Diabetes Mellitus
KCDC	Korea Centers for Disease Control and Prevention
KNHANES	Korea National Health and Nutrition Examination Survey
NCCP	National Cancer Control Program
NCSP	National Cancer Screening Program
WHO	World Health Organization
