# Supplementary material for: Determinants of Participation in the National Cancer Screening Program Among Older Korean Women: A Cross-Sectional Study Using Nationwide Population-Based Data
Source: Healthcare (Basel). 2026 Apr 15;14(8):1051. doi: 10.3390/healthcare14081051 (PMC13116162; doi:10.3390/healthcare14081051)
Supplement: Supplementary file 1 [file healthcare-14-01051-s001.zip › healthcare-4212028-supplementary.pdf]

【Questionnaire*】

1. Have you ever been married?

① Yes

② No

2. Where is your residence(town)?

① Dong (Urban)

② Eup/Myeon (Rura)

3. How far did you go in school? Or are you currently attending?

① Seodang/Classical Chinese Studies

② No Education

③ High School

④ 2-Year/3-Year College

⑤ Elementary School

⑥ 4-Year University

3-1. Did you finish that school? Please indicate whether you graduated.

① Graduated

② Completed Coursework

③ Currently Enrolled/On Leave of Absence

4. Adding up all income, including wages, real estate income, pensions, interest, government subsidies, and allowances from relatives or children, what is the approximate total household income for the past year? If it is difficult to answer with the annual income, please state the average monthly amount.

(Reference Period) ◎ Year ◎ Month

(Income Amount) | | | | | | 10,000 KRW

※ If no response is given, please wri

99999

5. Have you worked for at least one hour for income or worked as an unpaid family worker for at least 18 h during the past week? (This includes cases where you worked while on temporary leave, even if you are currently employed.)

① Yes

② No (→ To)

③ Under 15 years of age (Survey ended)

6. Which health insurance are you enrolled in?

① National Health Insurance (Regional)

② National Health Insurance (Employee)

③ Medical Aid

④ Not enrolled, Unknown

7. Have you enrolled in private medical insurance sold by insurance companies that subsidizes medical expenses, such as cancer insurance, cardiovascular disease insurance, or accident coverage insurance?

① Enrolled

② Not enrolled

③ Unknown

8. How would you rate your health?

① Very good

② Good

③ Average

④ Poor

⑤ Very poor

9-11. Mark V in the appropriate box.

	Diagnosed by a doctor
9. Hypertension	◻
10. Diabetes	◻
11. Dyslipidemia	◻

12. Do you usually engage in moderate-intensity sports, exercise, or leisure activities that cause you to be slightly out of breath or your heart to beat slightly fast for at least 10 min continuously?

① Yes

② No

13. The following is a question regarding your drinking experience over the past year. How often do you drink alcohol?

① Never in the past year (→ Return)

② Less than once a month

③ About once a month

④ 2-4 times a month

⑤ About 2-3 times a week

⑥ 4 or more times a week

14. Do you currently smoke regular cigarettes?

① Smoke every day

② Smoke occasionally

③ Smoked in the past, so do not smoke now

*Only research variables were extracted from the 8th Korea National Health and Nutrition Examination Survey(KNHANES VIII, 2019–2021) data.
